# Metabolomics of Two Pecan Varieties Provides Insights into Scab Resistance

**DOI:** 10.3390/metabo8040056

**Published:** 2018-09-23

**Authors:** Zhentian Lei, Clayton Kranawetter, Barbara W. Sumner, David Huhman, Daniel J. Wherritt, Andrew L. Thomas, Charles Rohla, Lloyd W. Sumner

**Affiliations:** 1 University of Missouri Metabolomics Center, Columbia, MO 652111, USA; sumnerb@missouri.edu; 2 Department of Biochemistry, University of Missouri, Columbia, MO 652111, USA; cdk374@mail.missouri.edu; 3 Noble Research Institute; Ardmore, OK 73402, USA; DVHUHMAN@noble.org (D.H.); ctrohla@noble.org (C.R.); 4 Department of Chemistry, University of Texas at San Antonia, San Antonia, TX 78249, USA; daniel.wherritt@utsa.edu; 5 Southwest Research Center, Division of Plant Sciences, University of Missouri, Mt. Vernon, MO 65712, USA; ThomasAL@missouri.edu

**Keywords:** metabolomics, UHPLC-MS, MS/MS, polyphenols, flavonoids, scab, pecan, *Carya*

## Abstract

UHPLC-MS-based non-targeted metabolomics was used to investigate the biochemical basis of pecan scab resistance. Two contrasting pecan varieties, Kanza (scab-resistant) and Pawnee (scab-susceptible), were profiled and the metabolomics data analyzed using multivariate statistics. Significant qualitative and quantitative metabolic differences were observed between the two varieties. Both varieties were found to have some unique metabolites. Metabolites that were only present or more abundant in Kanza relative to Pawnee could potentially contribute to the scab resistance in Kanza. Some of these metabolites were putatively identified as quercetin derivatives using tandem mass spectrometry. This suggests that quercetin derivatives could be important to pecan scab resistance.

## 1. Introduction

Pecan (*Carya illinoinensis*) belongs to the Juglandaceae family, and it is native to northeastern and central Mexico and southern United States. It is an economically important crop and has been cultivated commercially in 14 southern states ranging from North Carolina to California. The United States is the world’s second largest pecan producer, providing about half of the world’s pecans. Annual production of pecan in the U.S.A. totaled 293.85 million pounds in 2017 with a value of over $684 million [1].

The popularity and demand for pecan and other tree nuts have been increasing due to their tastes and health benefits [2,3,4,5,6]. It has been shown that frequent consumption of pecan provides protection against coronary heart disease and reduces death from heart attack [3,5,7,8]. This heart health-promoting effect of pecan is mainly associated with its high content of unsaturated fatty acids (>90%) and low content of saturated fatty acids. More recently, significant and dose-dependent inverse relationships have been reported between nut consumption and deaths from heart disease, cancer, and respiratory disease [8]. This could be due to the high antioxidant content found in pecan and other nuts. Pecan ranks top among the tree nuts in their antioxidant capacity [9]. Pecan antioxidants include tocopherols and polyphenols which have also been suggested to have anti-cancer properties [10,11,12].

Pecan scab disease, caused by the fungal plant pathogen *Fusicladium effusum*, is the single most destructive, economically significant, and widespread disease of pecans. Significant scab infection causes fusion of the developing endocarp (nut) to the pericarp (husk or shuck), resulting in the failure of the nut to mature properly and rendering the nuts unmarketable. The pathogen can also damage and destroy significant amounts of foliage, thereby reducing the photosynthetic capacity of the trees, especially critical during fruit ripening. Thus, scab significantly reduces pecan yields and quality. The pathogen can also infect other economically important tree nuts such as hickory (*Carya* spp.), and walnut (*Juglans* spp.). Scab is active throughout the growing season and is easily spread by wind and rain. It has been estimated that scab costs pecan growers at least $22 million each year in scab control costs and crop losses [13]. Moreover, repeated and widespread use of fungicides has resulted in the emergence of scab strains that are resistant to current fungicides [14,15]. Due to their toxic nature and the necessity for multiple repeated applications, there are growing concerns over the negative effects of fungicides on consumers and the environment. Highly scab-resistant pecan varieties normally do not require fungicide spray, thus reducing costs associated with crop loss and fungicide application.

Kanza and Pawnee are two pecan varieties widely planted in the southwestern USA such as Oklahoma and Texas. Kanza is rated moderately resistant to scab while Pawnee is considered moderately susceptible [16]. Understanding the mechanism underlying this differential resistance is critical to increasing our knowledge of scab resistance as well as developing strategies to prevent the disease and breeding for scab-resistant pecan varieties. Here, we report on the use of ultra-high performance liquid chromatography coupled to mass spectrometry (UHPLC-MS) based metabolomics to profile both Kanza and Pawnee leaves. Multivariate statistics across multiple years and geographical locations revealed significant qualitative and quantitative metabolic differences between these two varieties. Some polyphenols accumulated only in Kanza or were much more abundant in Kanza than in Pawnee. Some of these metabolites were tentatively identified as quercetin derivatives using tandem mass spectrometry (MS/MS). Their differential accumulation in Kanza suggests that they are involved in scab resistance.

## 2. Materials and Methods

### 2.1. General

Acetonitrile and water were LC-MS grade and obtained from Burdick and Jackson from Honeywell (Muskegon, MI, USA). Methanol was purchased from J. T. Baker (Phillipsburg, NJ, USA). Formic acid was LC/MS grade and obtained from Fisher Scientific (Hampton, NH, USA). Umbelliferone (7-hydroxycoumarin) and raffinose were purchased from Sigma-Aldrich (St. Louis, MO, USA).

Biological Samples: Pecan leaves were collected in triplicate from 3 individual trees per variety from the Samuel Roberts Noble Foundation McMillan pecan orchard (Ardmore, OK, USA) in June 2014 and August 2015. Fresh leaf samples were transported from the field over dry ice and then frozen immediately in liquid nitrogen upon arrival in the laboratory and stored at −80 °C. Additional samples were collected in triplicate or quadruplicate (three or four individual trees) from the University of Missouri Horticulture and Agroforestry Research Center (New Franklin, MO, USA) in September 2016, the University of Missouri Southwest Research Center (Mt. Vernon, MO, USA) in May 2017, and Kansas State University Pecan Experiment Field (Chetopa, KS, USA) in May 2017. Samples were handled in the same manner and stored at −80 °C until use.

### 2.2. UHPLC-QTOF-MS

Samples were lyophilized and ground to a fine powder. They were accurately weighed (10 mg ± 0.06 mg) and extracted with 1 mL of 80% methanol containing internal standard umbelliferone (18 µg/mL). The extracts from samples collected in 2014 and 2015 were then analyzed using a Waters Acquity UPLC system coupled to a QTOF Premier mass spectrometer (Waters). Separation of metabolites was achieved using a Waters Acquity 2.1 × 150 mm, 1.7 µm UPLC BEH C_18_ column and the following gradient: mobile phase B (acetonitrile) increased from 5% to 70% over 30 min, then to 95% in 3 min, held at 95% for 3 min, and returned to 95% mobile phase A (0.1% formic acid in water) for re-equilibration for 3 min. Mass spectral data were acquired from *m*/*z* 100 to 2000 in the negative electrospray ionization mode, with the nebulization gas at 850 L/h (350 °C) and the cone gas at 50 L/h (120 °C). Raffinose (*m*/*z* 503.1612) was used as a mass calibration reference compound in the independent lock-mass mode, with the lock mass scan (1 s) collected every 10 s for accurate mass measurements. The concentration of raffinose was 50 fmol/mL, and it was delivered at a flow rate of 0.2 mL/h. Tandem mass spectral data were acquired using a ramped collision energy from 10 to 60 eV.

Samples collected in 2016 and 2017 were analyzed on a Waters Acquity UPLC system coupled to a Bruker Maxis Impact QTOF mass spectrometer with the same UPLC conditions as above. Mass spectrometry was performed in the negative electrospray ionization mode with the nebulization gas pressure at 43.5 psi, dry gas of 12 L/min, dry temperature of 250 °C, and a capillary voltage of 4000 V. Auto MS/MS Mass spectral data were collected using the following parameters: MS full scan: 100 to 1500 *m*/*z*; number of precursors for MS/MS: 3; threshold: 10 counts; active exclusion: 3 spectra, released after 0.15 min; collision energy: dependent on mass, 35 eV at 500 Da, 50 eV at 1000 Da and 70 eV at 2000 Da. Mass spectra were calibrated using sodium formate that was co-infused in a calibration segment at the end of the gradient.

### 2.3. Data Processing and Statistical Analysis

Data acquired using the Waters QTOF Premier mass spectrometer were processed using Markerlynx v4.1 (Waters) to extract mass features (*m*/*z* and retention time pairs) with an intensity threshold of 150 and a mass window of 0.03. The mass features were normalized to an internal standard by dividing the abundance of the mass feature with that of the internal standard and then multiplying by 100. Putative annotations were made based on the accurate mass, MS/MS library search of our custom tandem spectral database [17], searches of online tandem spectra databases including Massbank [18], Massbank of North America (http://mona.fiehnlab.ucdavis.edu/), and the Global Natural Products Social Molecular Networking spectral library (https://gnps.ucsd.edu/ProteoSAFe/libraries.jsp). Putative annotations were also generated by manual spectral interpretation. Data collected with the Bruker Maxis Impact mass spectrometer were processed using DataAnalysis v4.2. Peak Dissect was performed using an S/N threshold of 3, max number of overlapping compounds: 5, cut-off intensity: 0.1%. The signal intensity was normalized to that of the internal standard umbelliferone (abundance of metabolite/abundance of umbelliferone × 100%) and used for statistical analysis. Multivariate statistical analyses such as principal component analysis (PCA) and ANOVA were performed using MetaboAnalyst [19]. 

## 3. Results and Discussions

This research aimed to determine if there could be a biochemical based mechanism for pecan scab resistance as observed in select scab-resistant varieties such as Kanza. Many plants can synthesize and accumulate specialized metabolites (also known as secondary metabolites or natural products) that protect themselves from pathogen attacks and environmental perturbations. Specialized metabolites that accumulate before (phytoanticipans) or that are induced by infection (phytoalexins) [20] can form the basis for nonhost resistance. These specialized metabolites include polyphenols, alkaloids, terpenoids, indoles, and glucosinolates. To ascertain if natural products are contributing to scab resistance, we used UHPLC-QTOF-MS to profile two contrasting pecan varieties, scab-resistant Kanza and scab susceptible Pawnee, in triplicate or quadruplicate across two years and multiple locations. The metabolomics data revealed significant differences between these two metabolic profiles.

### 3.1. Kanza and Pawnee Had Distinct Metabolic Profiles

**Figure 1:**
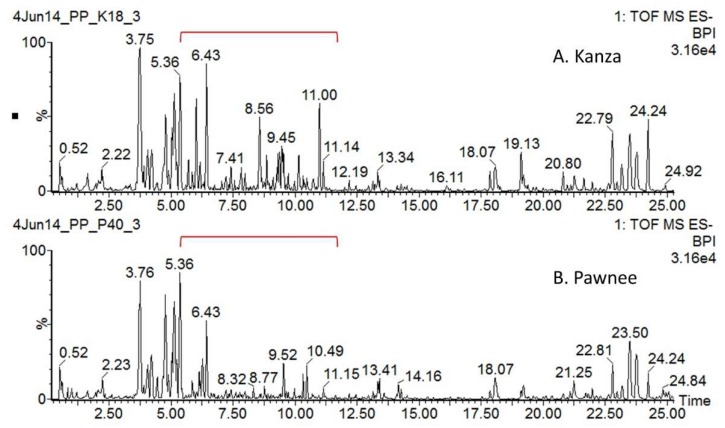
UHPLC-QTOF-MS base peak chromatograms of Kanza (**A**) and Pawnee (**B**) leaves. Dramatic differences were found between the Kanza and Pawnee metabolic profiles, particularly from 5 to 12 min outlined in red brackets. Numerous metabolites elute in this region, including phenolics and flavonoids.

**Figure 2:**
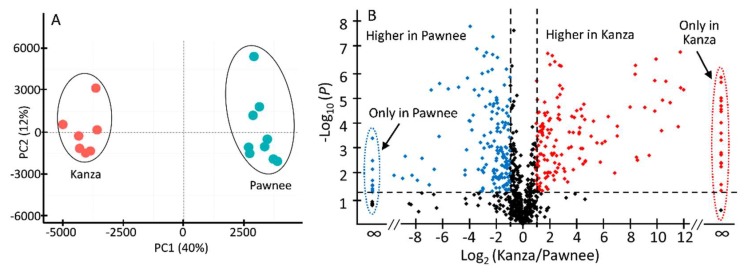
Principal component analysis (PCA) scores plot (**A**) and volcano plot (**B**) of all Kanza and Pawnee samples. The PCA scores plot shows a clear segregation of Kanza and Pawnee samples, indicating that Kanza and Pawnee have different metabolic profiles. A volcano plot of mass features (relative abundance >1) was constructed with *x*-axis presenting fold change (logarithms of Kanza-to-Pawnee ratios) and *y*-axis significance level (logarithms of *p* value). The plot shows the metabolites that were significantly higher in Kanza (upper right corner, red color) and in Pawnee (upper left corner, blue color). Two vertical dotted lines denote the boundary of two-fold change (Kanza relative to Pawnee, Log_2_ (0.5) = −1, Log_2_ (2) = 1) and the horizontal dotted line specifies the statistical significance cutoff (−Log (0.05) = 1.30).

Representative non-targeted UHPLC-QTOF-MS profiles of Kanza and Pawnee are shown in Figure 1. Substantial qualitative and quantitative differences were found between Kanza and Pawnee. One of the major conspicuous differences was highlighted in the chromatograms, i.e., the region from 5 to 11 min (Figure 1). Numerous specialized metabolites eluted in this region (i.e., 5–10 min), including phenolics, flavonoids and their glycosides [17]. The differences between Kanza and Pawnee metabolic profiles were also demonstrated by the principal component analysis (PCA) that showed a clear separation of Kanza from Pawnee (Figure 2A). This indicated that these two pecan varieties had distinct metabolic profiles, lending merit to the possibility of a differentially accumulated metabolite or metabolites contributing to scab resistance. Indeed, the volcano plot showed that Kanza differentially accumulated one set of metabolites while Pawnee accumulated a different set of metabolites (Figure 2B). The volcano plot was constructed using mass features with normalized abundance greater than 1 to minimize false positives derived from fragments and noise. The predominate metabolites (i.e., normalized abundance >5) that were significantly more abundant in Kanza or Pawnee were listed in Supplemental Tables S1 and S2, respectively. As shown in Table S1, some of these metabolites were exclusively accumulated in Kanza, and some were considerably higher in Kanza relative to Pawnee (i.e., the ratio of Kanza to Pawnee >100). Most of these metabolites were tentatively annotated as phenolics, flavonoids and their glycosides, based on their retention time and relative mass defect (RMD). Both retention time and RMD have been shown to be good quantifiers in metabolite annotation. More than 75% of these metabolites, (i.e., 113 out of 149) had a retention time that fell into the retention time range for phenolics, flavonoids and their glycosides (i.e., <20 min) [17]. RMD has been demonstrated to be a promising orthogonal parameter to differentiate plant specialized metabolite classes [21]. Phenolics and flavonoids typically have an RMD of less than 300 ppm. For example, flavone (IUPAC: 2-phenylchromen-4-one), the core structure of all flavonoids, has an RMD of 307 ppm. Oxidation of flavone, such as hydroxylation and glycosylation, decreases its RMD while methylation increases it (e.g., 141 ppm for 3,5,7,3′,4′,-pentahydroxyflavone, 325 ppm for 3,5,7,3′,4′,-pentamethylflavone, and 289 ppm for flavone-7-*O*-glucoside). In contrast, triterpene saponins and fatty acids have larger RMD values due to their relatively larger number of hydrogens and smaller number of oxygens. Their RMD values decrease as the degree of glycosylation increases. For example, β-amyrin has an RMD of 906 ppm, medicagenic acid 656 ppm, medicagenic acid diglucoside 526 ppm. The smaller RMD associated with mass features eluting before 20 min also strongly suggested that most of them were flavonoids and phenolics. Those eluting later in the gradient could be triterpene saponins or fatty acids as suggested by their retention times and relative mass defects.

**Figure 3:**
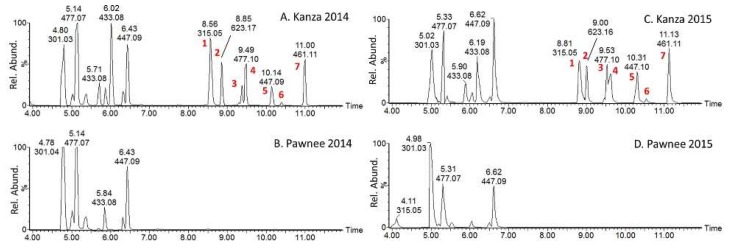
UHPLC-MS extracted ion chromatograms (4 to 12 min) of Kanza (**A**,**C**) and Pawnee (**B**,**D**). Samples A (Kanza) and B (Pawnee) were collected in 2014 while C (Kanza) and D (Pawnee) were collected in 2015 from the Noble McMillian Farm Pecan Orchard. The chromatograms were normalized to the highest peak in Kanza. Peaks labeled 1–7 were found mostly in Kanza regardless of sampling year and prioritized for peak annotation through MS/MS.

Plant metabolism is greatly influenced by biotic and abiotic stresses, which includes environmental impacts. Thus, it was necessary to demonstrate that the metabolic differences observed between Kanza and Pawnee were due to genetics rather than environmental differences because the leaf samples were collected from the field rather than from a carefully controlled environment such as a greenhouse. Towards this goal, samples were collected from the same trees during two different years. In a cross-comparison of the two varieties between consecutive years, the UHPLC-QTOF-MS metabolic profiles showed differences based upon both variety and sampling date (Figure S1). This indicated that the metabolic profiles were influenced not only by the variety but also by the sampling year. PCA analysis revealed a clear segregation of the samples based on the variety and the sampling year (Figure S2). It also revealed that the effect of sampling year on metabolic profiles was even greater than the cultivars as indicated by the PC1 along which the 2014 and 2015 samples were separated. This demonstrated the importance of using a carefully controlled environment in plant metabolomics and prompted us to investigate if the differences between Kanza and Pawnee observed in 2014 remained the same in 2015. The extracted ion chromatograms of some of the differentially accumulated mass features are provided for 2014 and 2015 (Figure 3). However, it was clear and shown in Figure 3, that these specific differences between Kanza and Pawnee were consistent between 2014 and 2015. To further increase the confidence level regarding the differences observed between Kanza and Pawnee, additional trees were sampled to further probe the influence of temporal and spatial (geographical) separation, i.e., Kansas and Missouri in 2016 and 2017. The specific differences between Kanza and Pawnee were consistent regardless of the sample year and locations (Figure S3). The differentially accumulated metabolites may contribute to Kanza’s scab resistance, and, thus were targeted for UHPLC-MS/MS analyses, UHPLC-MS-SPE (solid phase extraction) isolation [22], NMR analysis and future anti-scab activity assays.

### 3.2. Putative Metabolite Identification via MS/MS

**Figure 4:**
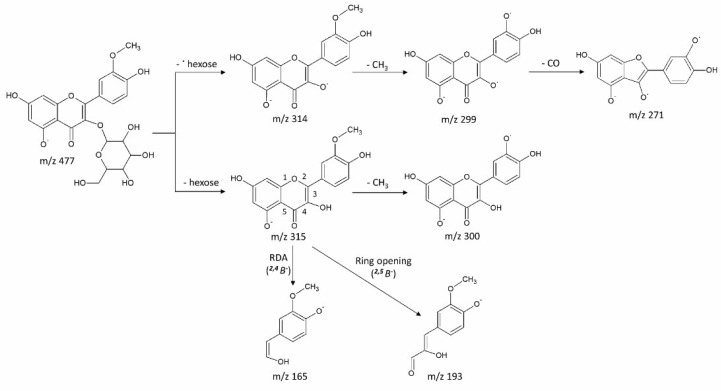
Proposed fragmentation of Peak 3 (9.38_477.1039). The fragmentation included loss of carbohydrate moiety, methyl group, and ring opening and re-arrangement. Fragments and their intensities are listed in Table 1.

Data-dependent MS/MS acquisition was performed on leaf extracts and used for metabolite identification. Metabolites that showed significant differences between Kanza and Pawnee were prioritized for MS/MS analysis (Figure 3, peak 1 to 7). Putative identifications were made by searching resultant tandem spectra against our custom tandem spectral database [17], an online tandem spectra database Massbank [18], Massbank of North America (http://mona.fiehnlab.ucdavis.edu/), and the Global Natural Products Social Molecular Networking spectral library (https://gnps.ucsd.edu/ProteoSAFe/libraries.jsp). Some of these metabolites (Figure 3, peak 3 and 4, peak 5 and 6) were identified as isomers as they had the same *m*/*z* values but different retention times. The results are summarized in Table 1, and a representative fragmentation scheme is proposed in Figure 4.

Peak 1 (8.56_315.0505, retention time_*m*/*z* value). The MS/MS spectrum displayed a fragment ion at *m*/*z* 300 [M-H-15]^−^, a characteristic loss of a methyl group (15 Da). This indicates that it was a methylated compound. The MS/MS spectrum was matched to that of an authentic standard rhamnetin (7-methylquercetin) (MassBank record number: PR100647) with a match score of 701.

Peak 2 (8.85_623.1634). Two predominant fragment ions at *m*/*z* 315 [M-H-308]^−^ and 314 [M-H-308-H^•^]^−^ were observed in the MS/MS spectrum. The loss of 308 suggests that the compound is di-glycosylated, probably a hexose (162 Da) plus a deoxyhexose (146 Da) moiety. Di-glycosylation is very common in plant natural products. The ion at *m*/*z* 315 could then be tentatively identified as the aglycone ion. The aglycone was methylated as evidenced by the ion at *m*/*z* 299 [M-H-308-15]^−•^, a loss of a methyl group (15 Da). The MS/MS spectrum was matched to the spectrum of authentic standard isorhamnetin-3-galactoside-6′′-rhamnoside (MassBank record number: PR100951) with a score of 949. Based on the results, the compound was tentatively identified as isorhamnetin-3-galactoside-6′′-rhamnoside (i.e., 3′-methoxyquercetin-3-galactoside-6′′-rhamnoside). 

Peak 3 (9.38_477.1039). The MS/MS spectrum revealed fragment ions at *m*/*z* 315 [M-H-162]^−^, *m*/*z* 314 [M-H-162-H^•^]^−^, 300 [M-H-162-15]^−^, 299 [M-H-162-H^•^-15]^−^, 271 [M-H-162-H^•^-15-28]^−^, 193 [M-H-162-122]^−^ and 165 [M-H-162-150]^−^. The data indicated the presence of a hexose moiety (loss of 162) and a methyl group (loss of 15 Da) as shown in Figure 4. The MS/MS spectrum was matched to that of rhamnetin-3-*O*-glucoside in the Global Natural Products Social Molecular Networking (GNPS) (Library Spectrum CCMSLIB00000847191) with a similarity score of 775 (Figure S4). However, lack of [M-H]^−^ and [M-H-2]^−^ pair suggested that the aglycone is most likely isorhamnetin rather than rhamnetin [17]. It was therefore identified as isorhamnetin-*O*-glucoside (3′-methylquercetin-*O*-glucoside). The proposed fragmentation pattern is shown in Figure 4. However, the glycosylation site could not be assigned confidently.

Peak 4 (9.49_477.1034). The compound had the same precursor ion and almost identical MS/MS spectrum as peak 3. It also matched to rhamnetin-3-*O*-glucoside with a score of 775. The close retention time and identical MS/MS spectrum suggest that these two metabolites are structural isomers, probably with different glycosylation sites. Tandem spectra of glycosylated flavonoids are typically dominated by the fragments from the flavonoid aglycone, making it difficult to differentiate glycosylated flavonoids derived from the same aglycone. Thus, it was tentatively identified as isorhamnetin-*O*-glucoside (3′-methylquercetin-*O*-glucoside). However, the glycosylation site could not be assigned confidently.

Peak 5 (10.14_447.0925). The MS/MS spectrum showed two fragment ions at *m*/*z* 447 [M-H]^−^ and 445 [M-H-2]^−^. This suggested that the compound has two vicinal hydroxyl groups such as rhamnetin, quercetin and luteolin [17] . The ion at *m*/*z* 315 [M-H-132]^−^ was an aglycone ion and matched to that of rhamnetin. The loss of 132 Da indicated that it was a pentoside. It was therefore tentatively identified as a rhamnetin-*O*-pentoside (7-methylquercetin-*O*-pentoside).

Peak 6 (10.39_447.0963). Two major fragments at *m*/*z* 315 [M-H-162]^−^ and *m*/*z* 314 [M-H-162-H^•^]^−^ were found in the MS/MS spectrum and determined to be aglycone ions. The loss of 162 indicted it was a pentoside. The aglycone was methylated as evidenced by a strong ion at *m*/*z* 299 [M-H-162-H^•^-15]^−^. The MS/MS spectrum was matched to isorhamnetin-3-*O*-glucoside (MassBank record number: PR040095) with a score of 681. It was, thus, tentatively identified as isorhamnetin-3-*O*-glucoside (3′-methylquercetin-3-*O*-glucoside).

Peak 7 (11.00_461.1071). The MS/MS spectrum displayed a base peak ion at 315 [M-H-146]^−^ and a strong fragment at *m*/*z* 314 [M-H-146-H^•^]^−^. The base ion peak could be determined as an aglycone ion. A strong ion at *m*/*z* 299 [M-H-146-H^•^-15]^−^ was also observed, indicating that the aglycone was methylated. The spectrum was matched to isorhamnetin-3-rutinoside (MassBank record number: PS091212) with a score of 805. However, the isorhamnetin-3-rutinoside (i.e., isorhamnetin-3-rhamnose-glucose) has an exact mass of 624.1690 Da, which was 162 Da (a hexose) higher than the observed ion. In addition, the data (loss of 146) suggested that it was a rhamnoside rather than a rutinoside. Thus, the compound was tentatively identified as methylated isorhamnetin-3-*O*- rhamnoside (methylated 3′-methylquercetin-3-*O*-rhamnoside).

Polyphenols have been documented to possess many biological activities including anti-fungal activity [23]. For example, increased concentrations of polyphenols such as flavonols, flavanols, dihydrochalcones, hydroxycinnamic acids, and anthocyanins were found in scab spots and surrounding tissues on apple skin, suggesting that these polyphenols were involved in defense responses against apple scab [24]. Flavan-3-ols such as quercetin have been reported to be particularly effective in protecting plants from fungal infections [25]. All seven compounds that were significantly higher in Kanza leaf extracts have been tentatively identified as methylated and/or glycosylated quercetin (5,7,3,3′,4′-pentahydroxyflavone) derivatives. They may contribute to scab resistance in Kanza. In fact, strong anti-scab activity was observed for methanolic extracts from Kanza leaves in a separate preliminary experiment (data not shown). The compounds will be isolated and purified using automated UHPLC-MS-SPE (solid phase extraction). The purified compounds will then be subjected to structural confirmation using NMR. Anti-scab assays will then be performed on the individual purified compounds.

## 4. Conclusions

LC-MS-based non-targeted metabolomics was used to profile leaves of two contrasting pecan varieties, scab-resistant Kanza and scab susceptible Pawnee in search of a biochemical basis for scab resistance in Kanza. Metabolomics data revealed that Kanza and Pawnee have distinct metabolic profiles. One of the significant differences between the two profiles was in the region where polyphenols typically eluted under the current experimental conditions. The difference was consistent in samples collected from multiple geographical locations and in different years. This indicated that the differences (unique compounds in Kanza) were due to genetics rather than environment, and may result in the stable (or consistent) scab resistance seen in Kanza. It has been established that plants produce two different types of defensive compounds, i.e., phytoalexins and phytoanticipins [20]. Phytoalexins are produced in plant tissues in response to pathogen infection while phytoanticipins are synthesized constitutively in plants and increase upon infection. As these compounds were found in leaves free of infection, they are considered as phytoanticipins. Profiling infected leaves may reveal additional phytoalexins that are also important to scab resistance. The compounds were tentatively identified as quercetin derivatives which have previously shown anti-microbial activity towards other fungal pathogens. Their structures and anti-scab activity will be ascertained using UHPLC-SPE-MS, NMR, and anti-fungal assays. The resultant information can then be used for breeding or engineering of scab-resistant pecan variety in the future.

## Figures and Tables

**Table 1:** Putative identification of peaks significantly accumulated in Kanza leaves using UHPLC–MS/MS in negative electrospray ionization mode. The MS/MS spectra were acquired using a ramped collision energy from 10 to 60 eV. The identifications were based on MS/MS spectral data and spectral library matching.

No	tR min	[M-H]^−^	Formula	Error ppm	MS/MS, *m*/*z* (intensity %)	Identification
1	8.56	315.0505	C16H12O7	0.06	315 (70), 300 (41), 299 (14), 271 (40), 243 (12), 165 (100), 121 (50)	rhamnetin (7-Methoxyquercetin)
2	8.85	623.1634	C28H32O16	3.5	623 (100), 314 (85), 299 (20), 191 (20)	Isorhamnetin-3-galactoside-6′′-rhamnoside
3	9.38	477.1039	C22H22O12	1.3	477 (58), 315 (70), 314 (100), 300 (12), 299 (32), 271 (10), 193 (9), 165 (12)	Isorhamnetin-3-*O*-glucoside
4	9.49	477.1034	C22H22O12	0.2	477 (58), 315 (70), 314 (100), 300 (12), 299 (32), 271 (10), 193 (9), 165 (12)	Isorhamnetin-3-*O*-glucoside
5	10.14	447.0925	C21H20O11	0.4	447 (100), 445 (80), 432 (30), 316 (40), 315 (40), 161 (40), 101(60)	Rhamnetin-*O*-pentoside
6	10.39	447.0963	C21H20O11	8	447 (35), 315 (100), 314 (80), 299 (60), 271 (20), 165 (15)	Isorhamnetin-3-*O*-glucoside
7	11.02	461.1071	C22H22O11	2.6	461 (71), 315 (100), 314 (80), 299 (70), 271 (30), 165 (28)	Methylated isorhamnetin-3-*O*-rhamnoside

## Supplementary Materials

The following are available online at http://www.mdpi.com/2218-1989/8/4/56/s1, Table S1. List of mass features more abundant in Kanza. Table S2. List of mass features more abundant in Pawnee. Figure S1: Representative base peak chromatograms of Kanza and Pawnee leaves collected in 2014 and 2015. Figure S2: PCA plot of Kanza and Pawnee leaves collected in 2014 and 2015. Figure S3: Extracted ion chromatograms of Kanza and Pawnee leaves collected in 2017 from three different orchards. Figure S4: Representative MS/MS spectrum library searching results.
