# Supplementary figures and images for: Metabolomics of Two Pecan Varieties Provides Insights into Scab Resistance
Source: Metabolites. 2018 Sep 23;8(4):56. doi: 10.3390/metabo8040056 (PMC6315810; doi:10.3390/metabo8040056)

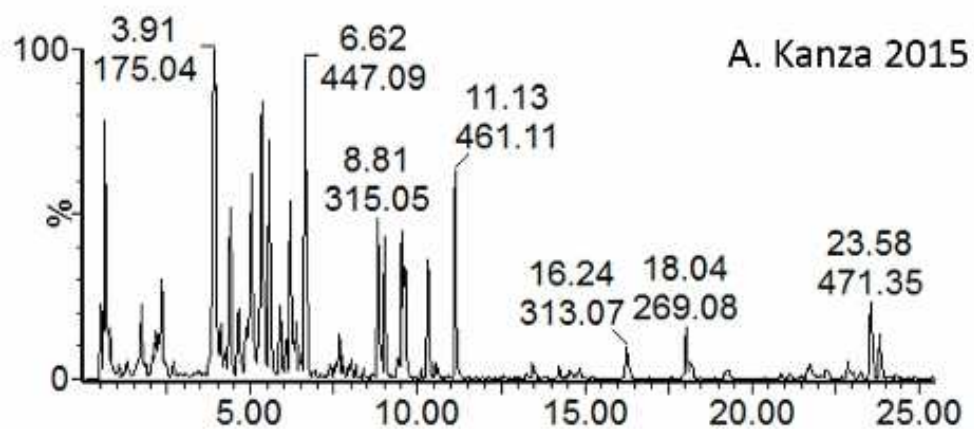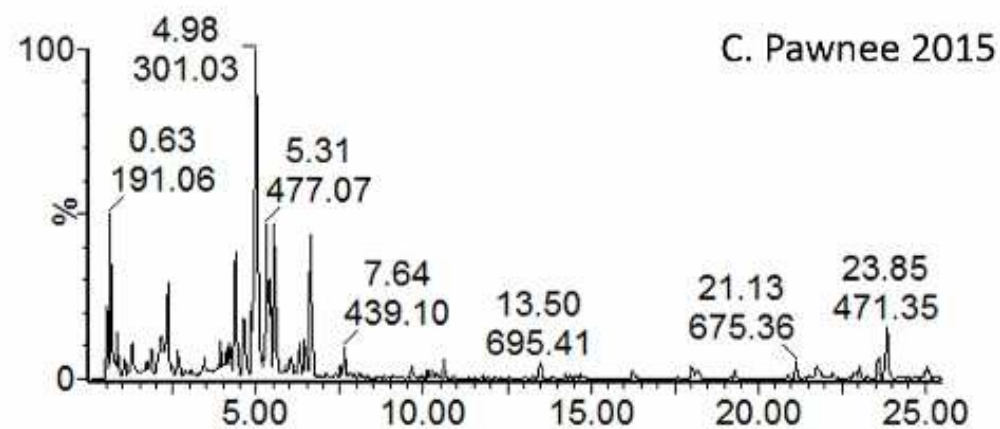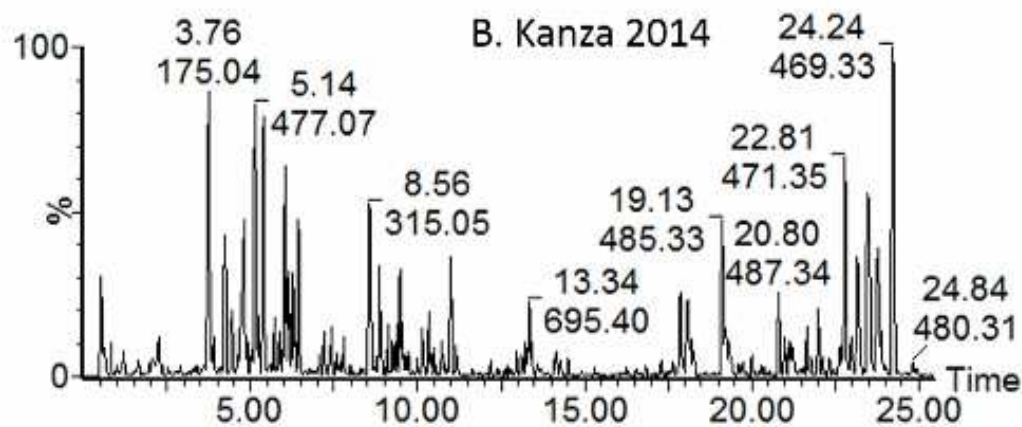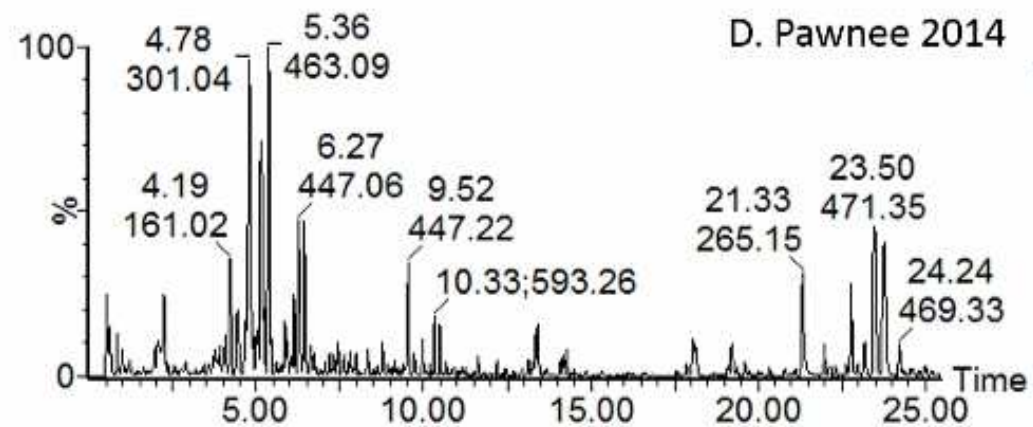

Supplement: Supplementary file 1 [file metabolites-08-00056-s001.zip › Figure S1.pdf]

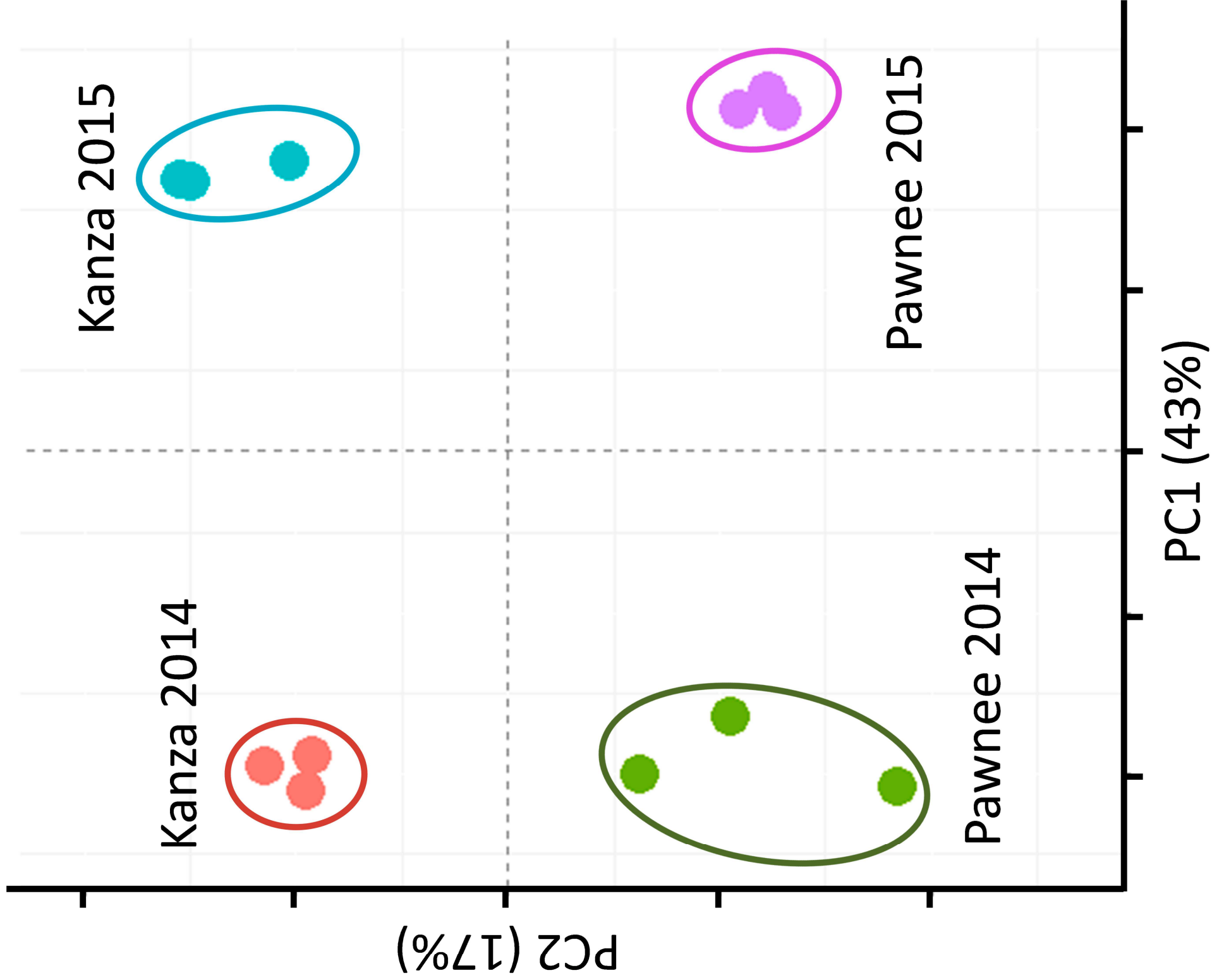

Supplement: Supplementary file 1 [file metabolites-08-00056-s001.zip › Figure S2.pdf]

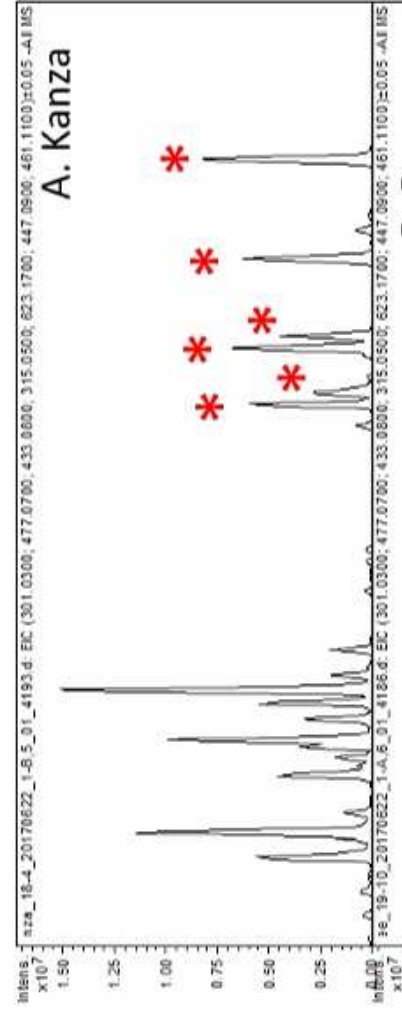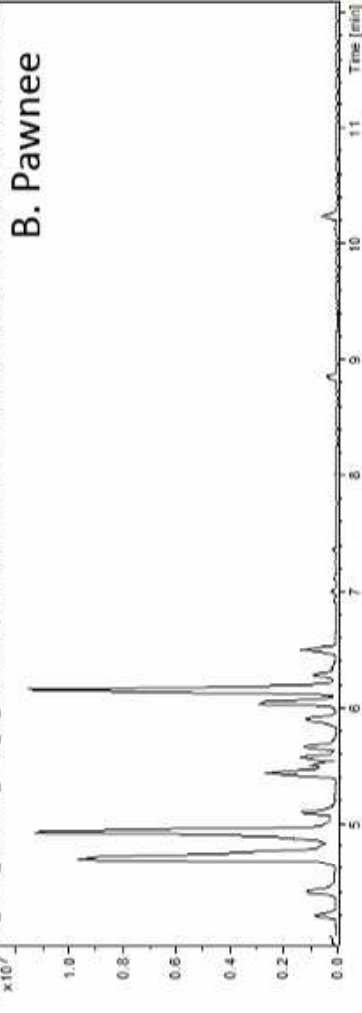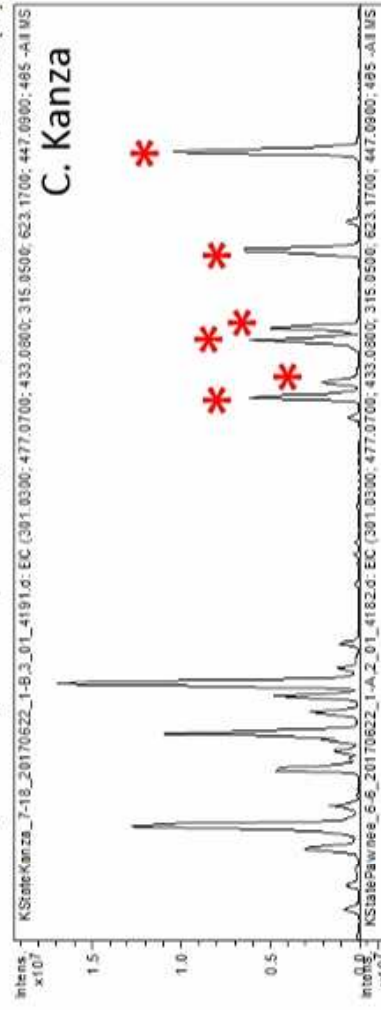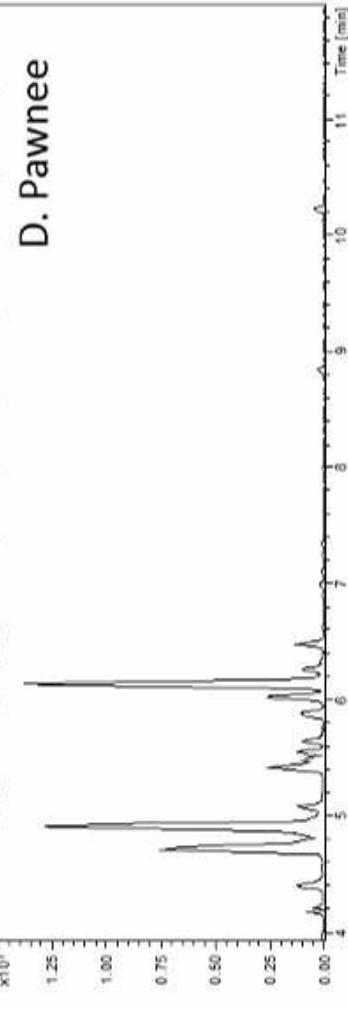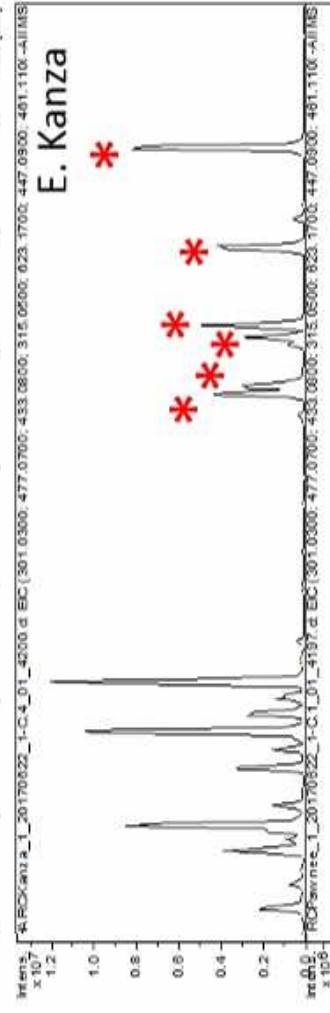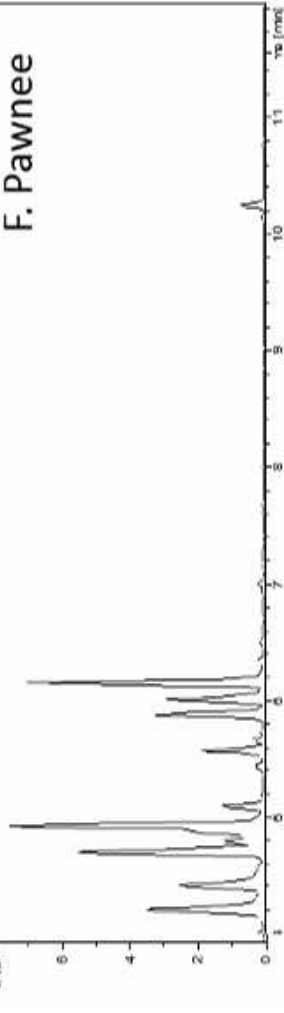

Supplement: Supplementary file 1 [file metabolites-08-00056-s001.zip › Figure S3.pdf]

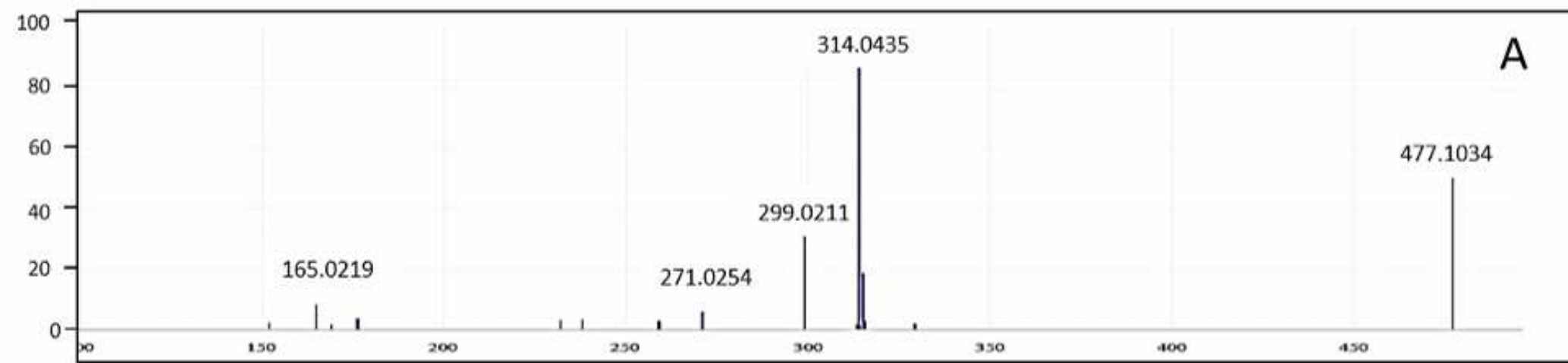

18Aug15\_K20\_DDA\_MSMS 418 (9.650)

2: TOF MSMS 475.01ES-

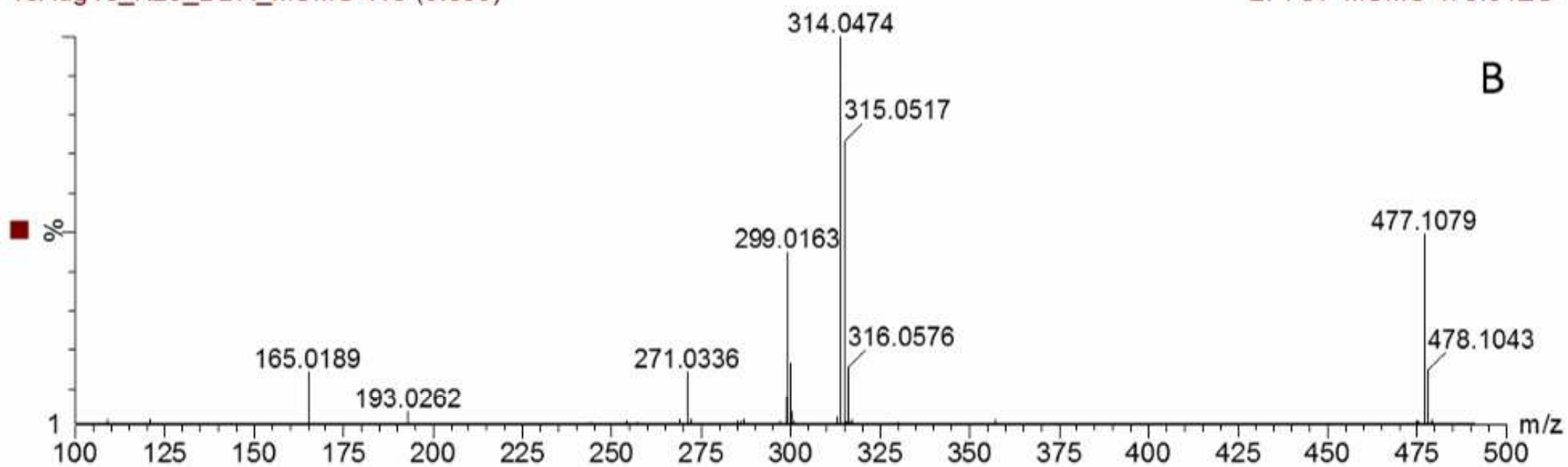

Supplement: Supplementary file 1 [file metabolites-08-00056-s001.zip › Figure S4.pdf]
